# Integration of transcriptomics reveals ferroptosis-related signatures and immune cell infiltration in bronchopulmonary dysplasia

**DOI:** 10.1016/j.heliyon.2023.e21093

**Published:** 2023-10-20

**Authors:** Zhengyun Hu, Chong Liu, Yan Mao, Jianwei Shi, Jinwen Xu, Guoping Zhou, Feng Jiang

**Affiliations:** a Department of Pediatrics, Songjiang Hospital Affiliated to Shanghai Jiao Tong University School of Medicine (Preparatory Stage), Shanghai, China; b Department of Pediatrics, The First Affiliated Hospital of Nanjing Medical University, Nanjing, China; c Department of Neurosurgery, Xuanwu Hospital, Capital Medical University, Beijing, China; d Department of Pediatric Nephrology, Wuxi Children's Hospital, Wuxi, China; e Department of Neonatology, Obstetrics and Gynecology Hospital of Fudan University, Shanghai, China

**Keywords:** Bronchopulmonary dysplasia, Diagnostic, Ferroptosis, Immune infiltration, Prediction model

## Abstract

Ferroptosis has emerged as a significant factor in the development of bronchopulmonary dysplasia (BPD). Nevertheless, our understanding of the potential involvement of ferroptosis-related genes (FRGs) in BPD remains incomplete. In this study, we leveraged the Gene Expression Omnibus (GEO) database to investigate this aspect. We identified 20 differentially expressed FRGs that are associated with BPD, shedding light on their potential role in the condition.LASSO along with SVM-RFE algorithms found that 12 genes: MEG3, ACSL1, DPP4, GALNT14, MAPK14, CD82, SMPD1, NR1D1, PARP3, ACVR1B, H19, and SLC7A11 were closely related to ferroptosis modulation and immunological response. These genes were used to create a nomogram with good predictive power and were found to be involved in BPD-linked pathways. In addition, the marker genes-based prediction model performed well in external validation data sets. The study also showed a significance between BPD and control samples in terms of immune cell infiltration. These findings may help improve our understanding of FRGs in BPD and lead to the development of more effective immunotherapies.

## 1 Introduction

Bronchopulmonary dysplasia (BPD) is one of the major neonatal diseases that threaten the survival and prognosis of premature infants [1]. Immature lung, acute injury of lung tissue, and abnormal repair of lung tissue after injury are the three causes of BPD [2]. Alveolar simplification and pulmonary angiogenesis disorders are the main pathological basis of BPD [3]. Postnatal management of respiratory failure with invasive mechanical ventilation (IMV) and oxygen use in the neonatal intensive care unit further leads to inflammation and maladaptive lung development [4]. BPD is a complex multifactorial disorder of prematurity, and its pathogenesis involves a large number of intrauterine and extrauterine factors [5]. The incidence of BPD rises as more premature babies survive [6]. Many non-drug and pharmacological interventions have been studied for the prevention and management of BPD, however, there is still no clear consensus on the use of early diagnostic tests and precise drugs for BPD [[7], [8], [9]].

Ferroptosis belongs to a novel type of iron-dependent programmed cell death resulting from lipid peroxidation (LPO), which is different from other programmed cell death in morphological and biochemical features [10]. Its major morphological features contain mitochondrial narrowing as well as elevated density of the mitochondrial membrane, along with the decrease or disappearance of mitochondrial crystals and disintegration of the external membrane [11]. The relevant biochemical manifestations are iron ions accumulation, reduced cysteine absorption as well as glutathione synthesis (GSH), activation of the mitogenic stimulated protein kinase system, and release of arachidonic acid [12]. Many reports have uncovered that ferroptosis harbors various functions in many diseases, such as neurologic system disorders, lung diseases, autoimmune diseases, heat stress diseases as well as cancers [[12], [13], [14], [15], [16]].

There is a growing body of evidence suggesting that ferroptosis plays a crucial role in respiratory disorders characterized by inflammation. These disorders include conditions such as acute lung injury and acute respiratory distress syndrome (ARDS) [[17], [18], [19]]. In the meantime, ferroptosis is directly related to BPD pathogenesis. Qiang discovered that the decrease in NRF2 suppresses STAT3 as well as SLC7A11 expression to induce ferroptosis and aggravate oxygen and glucose deprivation/reperfusion (OGD/R)-induced lung damage [20]. Pulmonary expression of SLC7A11 is increased in hyperoxia exposure-induced mouse model of BPD [21]. MAPK14 is downregulated in lungs of mouse model of BPD [22]. Downregulated H19 relieves pulmonary injury and reduced inflammatory response in BPD newborn mice [23]. Ferroptosis in alveolar epithelial cells may promote ALI development by mediating iron accumulation. Lipopolysaccharide (LPS) induced iron-dependent ferroptosis in BEAS-2B cells by inhibiting the Keap1-Nrf2/HO-1 pathway, resulting in severe lung damage with inflammatory cell infiltration [24].

In this study, machine learning algorithms was utilized for analysis and verification of the accuracy of FRGs as biological markers for BPD. In addition, bioinformatics was utilized to investigate the association of FRGs and the immune microenvironment and drugs. The current research may provide novel information on the physiopathology and treatment of BPD.

## 2 Materials and methods

### 2.1 Data collection together with handling

Gene expression data for individuals with BPD and control subjects were obtained from the Gene Expression Omnibus (GEO) database. Specifically, we utilized the GSE32472 dataset, which includes microarray data derived from blood samples collected from newborns diagnosed with BPD. This dataset encompasses gene expression analyses conducted at three time points: on the 5th, 14th, and 28th days of their lives. This dataset comprised data from 112 control subjects and 187 BPD patients. As an external validation dataset, GSE8586 included 34 control samples and 20 BPD umbilical cord tissue samples from infants. The adequacy of the sample size was assessed by calculating the d-effect size using G*Power [25], affirming its statistical reliability. For the identification of FRGs, we queried the FerrDb V2 database (accessible at http://www.zhounan.org/ferrdb/current/), extracting data from three gene categories: drivers, suppressors, and markers [26]. This process yielded a collection of 420 FRGs (as detailed in Table S1). Furthermore, to validate the expression patterns of hub genes in lung tissues affected by hyperoxia-induced BPD, we utilized two additional datasets, namely GSE197403 and GSE25286, which featured data from both hyperoxia-induced BPD subjects and control subjects.

### 2.2Analysis of differential genes expression

From the GSE32472 dataset, we extracted the expression profiles of 420 FRGs, encompassing both BPD and control samples. To identify the genes that exhibited differential expression between these two groups, we conducted a Wilcoxon test. Statistical significance was established at p < 0.05, indicating genes that were differentially expressed and were termed DE-FRGs.

### 2.3 Analysis of enhanced enrichment annotations

To assess the biological significance of the DEGs, we employed the Metascape database [27], which can be accessed at http://metascape.org. Using this platform, we conducted Gene Ontology (GO) and Kyoto Encyclopedia of Genomes (KEGG) pathway enrichment analyses on the specific genes of interest. To ensure robust results, we set the criteria for statistical significance as follows: a minimum overlap of three genes and a p-value cut off of 0.01.

### 2.4 Identification of optimal BPD diagnostic gene biomarker

To identify genetic markers and potential biological markers for BPD, we began by selecting the DE-FRGs from both control and BPD samples. To reduce dimensionality and select the most relevant features, we employed the LASSO algorithm. Subsequently, we built SVM-RFE models. We compared the mean rates of judgment error through a 10-fold cross-validation process. The goal was to determine the optimal genetic biological markers for BPD by leveraging the outcomes from these two overlapping algorithms. To evaluate the diagnostic potential of these optimal genetic biomarkers, we generated ROC curves and calculated essential metrics such as the AUC, sensitivity, specificity, and precision. Additionally, we employed the predictive function to develop a model based on the twelve marker genes. ROC curves were utilized to assess the diagnostic accuracy and power of this logistic regression model. This comprehensive approach allowed us to pinpoint potential genetic markers and assess their diagnostic utility in identifying individuals with BPD.

### 2.5 Independent validation assessment

We conducted ROC analyses to evaluate the discriminative capacity of the predictive model in distinguishing between individuals with BPD and non-BPD controls, utilizing the GSE8586 dataset. To visualize the ROC curves, we utilized the "pROC" R package. This analysis aimed to assess the model's performance in correctly classifying BPD and non-BPD subjects based on its predictive power.

### 2.6Verification of hub genes in external datasets

The GSE197403 and GSE25286 dataset included expression data for the hub genes in both BPD and control samples. To identify differentially expressed hub genes, we performed the Wilcoxon test using R software. Statistical significance was assessed using a p-value threshold of less than 0.05.

### 2.7 GSEA analysis

We conducted Gene Set Enrichment Analysis (GSEA) by using the "clusterProfiler" and "enrichplot" R packages. To achieve this, we assessed the relationships between these marker genes and all other genes in the dataset. To do this, we ranked the genes based on their correlations with the marker genes, sorting them from the highest to lowest correlation values. Subsequently, the classified genes were assigned to a set for testing. The predefined KEGG pathway set was employed to determine the abundance of these pathways within the gene set. This process allowed us to gain insights into the pathways that were significantly associated with the twelve marker genes in the context of BPD.

### 2.8 GSVA analysis

For this analysis, we employed the R package "GSVA." The basis for our analysis was the KEGG enrichment assembly applied to each of the marker genes. We employed the "limma" package to compare GSVA scores between two groups: one characterized by high expression levels of the marker genes and the other by low expression levels. Our screening criteria were defined as follows: a p-value <0.05 and |t| > 2. When the t-value exceeded zero, it indicated an enrichment of the pathway in the group with high marker gene expression. Conversely, when the t-value fell below zero, it indicated significant enrichment in the group with low marker gene expression. This analysis enabled us to identify pathways associated with varying expression levels of the marker genes and their potential relevance to BPD.

### 2.9 Determination of nomogram

We generated a diagnostic nomogram using the "rms" and "rmda" R packages, based on the selected candidates from FRGs. To assess the reliability of our predictions and their alignment with actual outcomes, we used a calibration curve. Additionally, we conducted a Decision Curve Analysis (DCA) to evaluate whether the decisions made by our model were supportive of patient care. This analysis helps determine the clinical utility of the model and whether it provides meaningful guidance for decision-making in the context of diagnosing or managing BPD.

### 2.10 Immune infiltration analysis

CIBERSORT is a technique employed to estimate the relative proportions of various cell types within complex tissues, primarily relying on patterns of gene expression [28]. In our study, we utilized CIBERSORT to analyze the relative proportions of 22 distinct types of infiltrating immune cells in each sample. This analysis provided valuable insights into the composition of immune cells in the studied samples. To simplify the analysis, we aggregated the fractions of the various immune cell types into a single value for each sample.

### 2.11 Identification of the potential drugs

We utilized the Drug Gene Interaction Database (DGIdb) [29], accessible at https://dgidb.genome.wustl.edu/, as a valuable resource for investigating potential interactions between drugs or molecular compounds and the FRGs. Through DGIdb, we analyzed the potential drugs or compounds that might interact with FRGs. Subsequently, we visualized the drug-gene interaction network using the Cytoscape software, allowing us to gain a comprehensive understanding of the interactions between these FRGs and potential therapeutic agents.

### 2.12 In vitro experiments

We obtained human type II alveolar lung epithelium cells, specifically A549 cells, from the American Type Culture Collection (ATCC). These cells were cultured in a growth medium comprising 10 % fetal bovine serum (FBS) and Dulbecco's Modified Eagle Medium (DMEM), which was supplemented with a 1 % penicillin/streptomycin solution. For our hyperoxia experiments, we utilized a modular incubation chamber known as the MIC-101, manufactured by Billirups-Rothenberg Inc. in Del-Mar, CA. We followed the manufacturer's instructions carefully to create the experimental conditions required for our study. Specifically, the hyperoxia group of cells was cultured within the MIC-101 incubator, which maintained an environment containing 85 % oxygen (O_2_) and 5 % carbon dioxide (CO_2_). In contrast, the normoxia group continued to be cultured in a standard incubator with an atmosphere composed of 21 % O_2_ and 5 % CO_2_. To extract total RNA from the cells, we utilized the RNeasy Mini Kit, which is manufactured by Qiagen, Hilden, Germany. The extracted RNA was then subjected to reverse transcription into complementary DNA (cDNA) using ReverTra Ace, a product from Toyobo in Osaka, Japan. For real-time quantitative PCR analysis, we employed the LightCycler 480 system, manufactured by Roche Applied Science, Indianapolis, IN. The SYBR Premix Ex *Taq*II kit, produced by Takara in Japan, was used for this purpose. The primers utilized in the PCR assays were designed based on PrimerBank and synthesized by Generay in Shanghai, China. These procedures allowed us to assess and quantify gene expression levels in the A549 cells under different experimental conditions. The sequences were as follows: DPP4, forward 5′-TACAAAAGTGACATGCCTCAGTT-3′ and reverse 5′- TGTGTAGAGTATAGAGGGGCAGA-3’; CD82, forward 5′- TGTCCTGCAAACCTCCTCCA-3′ and reverse 5′- CCATGAGCATAGTGACTGCCC-3’; MAPK14, forward 5′- TCAGTCCATCATTCATGCGAAA-3′ and reverse 5′- AACGTCCAACAGACCAATCAC-3’; ACVR1B, forward 5′- CAGGATCGACTTGAGGGTGC-3′ and reverse 5′- CGGCGATGATGCCTACCAG-3’; ACSL1, forward 5′- CGACGAGCCCTTGGTGTATTT-3′ and reverse 5′- GGTTTCCGAGAGCCTAAACAA-3’; PARP3, forward 5′- GCCCTGGGTACAGACTGAG-3′ and reverse 5′- CGCTTCTCTGCGGGTATGG-3’; and GAPDH, forward 5′- TGTGGGCATCAATGGATTTGG-3′ and reverse 5′- ACACCATGTATTCCGGGTCAAT -3’.

### 2.13 Statistical analysis

We employed the Wilcoxon test to compare the mean values between the BPD and control groups. To assess the relationships among the DE-FRGs, we conducted Pearson's correlation analysis. Venn diagrams were created using the "VennDiagram" package to visualize the intersections and differences among different gene sets or groups. We visualized the drug-gene interaction network using Cytoscape version 3.8.2, allowing us to gain a comprehensive understanding of the interactions between drugs or compounds and genes. For all analyses, a significance level of p < 0.05 was considered statistically significant. The R package (V4.2.1) was utilized for conducting these analyses.

## 3 Results

### 3.1 Recognition of DE-FRGs in GSE32472

A comprehensive examination of the gene expression profiles of the 420 FRGs in the GSE32472 dataset, contrasting the BPD and control samples, is available in Table S2. Additionally, we conducted an in-depth characterization of the top 20 DE-FRGs based on the criteria of |logFC| > 0 and P < 0.001. These analyses provided valuable insights into the differential expression patterns of these genes between the two groups. These genes were visually represented in a clustering heatmap, as shown in Fig. 1A. Fig. 1B illustrated the expression patterns of these top 20 DE-FRGs between the BPD and control samples using a columnar scatter plot, providing a clear visualization of the differences in gene expression between the two groups. To further investigate whether FRGs have a key part in BPD progression, correlation research was conducted among these DE-FRGs. A part of the FRGs exhibited strong synergistic impacts. The gene interaction network map provided additional verification of the link between these DE-FRGs (Fig. 1C).

**Fig. 1:**
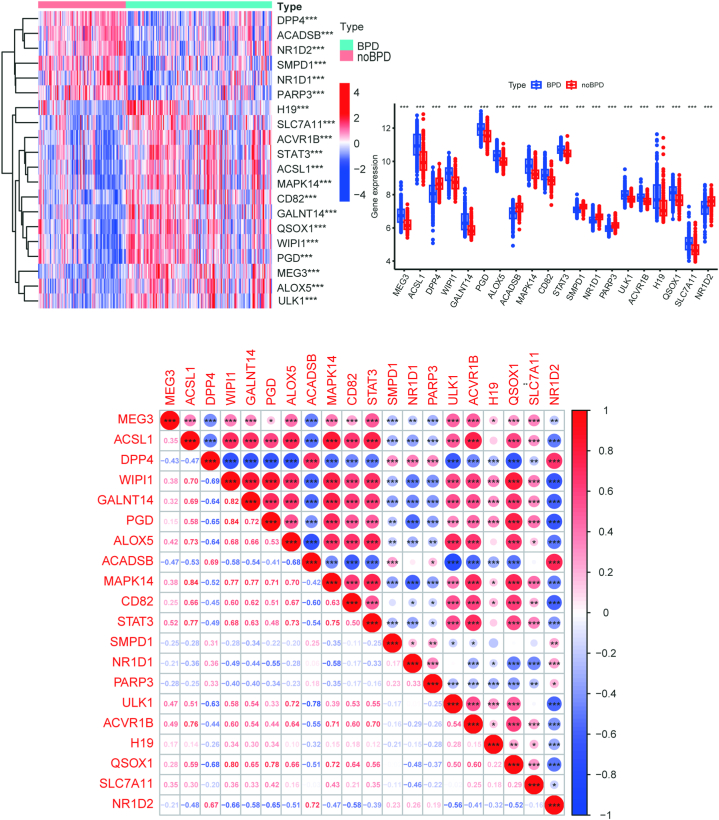
**Expression levels of DE-FRGs in** GSE32472 **dataset** (A) The heatmap visually illustrates the expression patterns of DE-FRGs. (B) The columnar scatter plot depicts the expression levels of the top 20 DE-FRGs in both the BPD and control samples. (C) The correlation plot reveals the associations between the top 20 DE-FRGs within the context of BPD. The color scheme indicates correlations, with blue representing negative correlations and red representing positive correlations. (*p < 0.05, **p < 0.01, ***p < 0.001).

### 3.2 Functional enrichment analysis

**Fig. 2:**
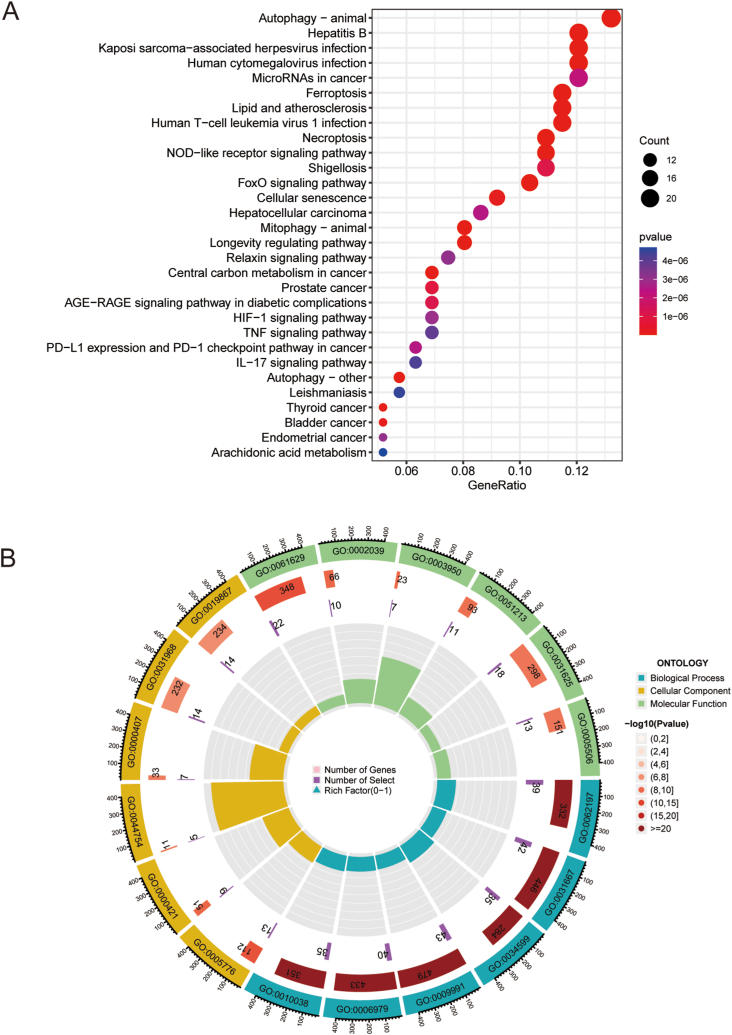
**Functional analysis of DE-FRGs** (A) Bubble plot of KEGG enrichment analyses based on DE-FRGs. (B) The circle plot showing the findings of GO enrichment analysis.

The GO terms and KEGG pathways were visualized using Metascape. The most prominent KEGG and GO pathways identified included ferroptosis, modulation of autophagy, HIF-1 signaling pathway, response to oxidative stress, iron ion binding, and modulation of interleukin-17 production, etc. These results, illustrated in Fig. 2A–B, suggest that DE-FRGs may play a role in the pathogenesis of BPD.

### 3.3 Recognition of 12 DE-FRGs as diagnostic genes for BPD

**Fig. 3:**
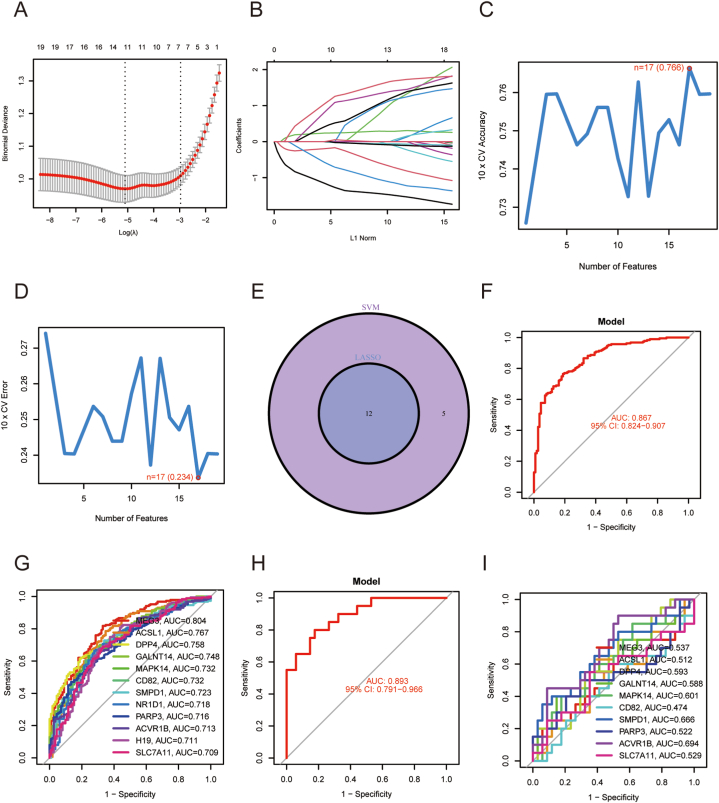
**Twelve DE-FRGs were identified as biomarkers for BPD** (A, B) The LASSO algorithm, with a penalty setting and 10-fold cross-validation, was employed to identify 12 markers associated with BPD. (C, D) The SVM-RFE algorithm was used to filter the 20 DE-FRGs and identify the optimal combination of marker genes, resulting in the identification of 17 optimal marker genes. (E) The marker genes derived from both the LASSO and SVM-RFE models were combined for further analysis. (F) We developed a logistic regression model to determine the AUC for BPD sample classification in the GSE32472 dataset. (G) ROC curves were generated for the 12 biomarkers in the GSE32472 dataset. (H) Another logistic regression model was created to calculate the AUC for BPD sample classification in the GSE8586 external validation dataset. (I) ROC curves were generated for the 10 biomarkers in the GSE8586 dataset.

We selected twelve markers associated with BPD using LASSO regression with a 10-fold cross-validation penalty setting, as depicted in Fig. 3A–B. Subsequently, we utilized the SVM-RFE algorithm to screen the 20 DE-FRGs. This process resulted in the discovery of 17 optimal characteristic genes, with a maximum accuracy of 0.766 and a minimal RMSE of 0.234, as indicated in Fig. 3C–D. Among these genes, twelve marker genes (MEG3, ACSL1, DPP4, GALNT14, MAPK14, CD82, SMPD1, NR1D1, PARP3, ACVR1B, H19, and SLC7A11) were identified for further investigation. These marker genes were selected based on their intersection, as shown in Fig. 3E. We constructed a regression model using the glmnet package in R, utilizing these 12 marker genes as the basis. Subsequent ROC curves demonstrated that the model effectively differentiated BPD samples from control samples, with an AUC value of 0.867 (Fig. 3F). Moreover, ROC curves were employed to assess the individual genes' associations with BPD, with each gene achieving an AUC exceeding 0.7 (Fig. 3G). To further validate the diagnostic potential of the constructed DE-FRGs, we included an external BPD dataset named GSE8586 from the GEO database in our analysis.The ROC curves of BPD patients showing the model and 10 individual DE-FRGs in the testing set were shown in Fig. 3H and I, confirming that the DE-FRGs model was a good predictive factor for BPD.

**Fig. 4:**
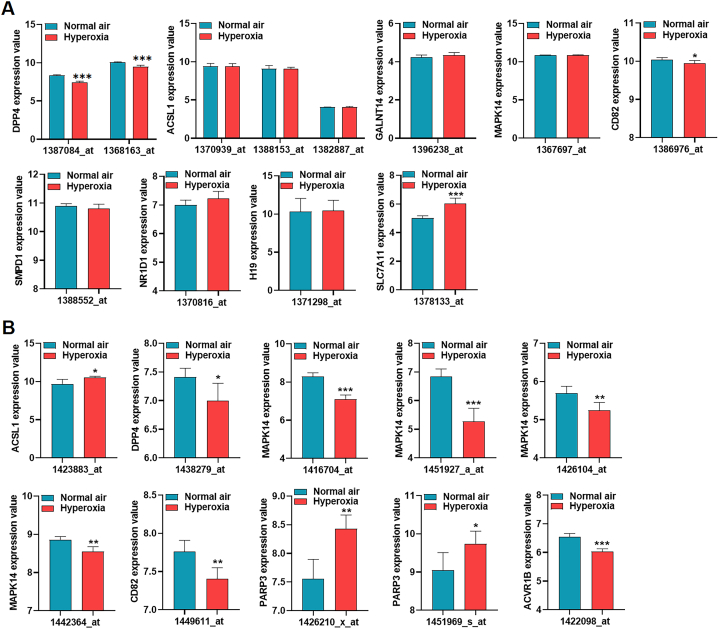
**Expression validation** of DE-FRGs in BPD The expression values of hub genes in the model based on the GSE197403 (A) and GSE25286 (B) datasets.

The GSE197403 dataset included ACSL1, DPP4, GALNT14, MAPK14, CD82, SMPD1, NR1D1, PARP3, H19, and SLC7A11 genes. Among them, DPP4, CD82, and SLC7A11 genes showed statistically differential expression in lungs between the control group and the BPD group (Fig. 4A). The GSE25286 dataset included all 12 genes, and statistical outputs of expression values of genes in the model were provided in Table S3. ASCL1, DPP4, MAPK14, CD82, PARP3, and ACVR1B showed statistically differential expression in lungs between the control group and the BPD group (Fig. 4B).

To further validate the significance of the six marker genes in the context of BPD, we conducted experiments using A549 cells. These cells were cultured in a specialized incubator with an environment consisting of 85 % O_2_ and 5 % CO_2_ to mimic the conditions associated with BPD. In contrast, control cells were cultured in an incubator with a standard atmosphere containing 21 % O_2_ and 5 % CO_2_.

The results obtained from RT-qPCR, as presented in Fig. S1, demonstrated that DPP4, CD82, MAPK14, and ACVR1B exhibited downregulation in the hyperoxia group when compared to the normoxia group. These findings confirm the involvement of these genes in the response to hyperoxia, which is a significant aspect of BPD pathogenesis. In contrast, ACSL1 and PARP3 exhibited elevated expression levels in the hyperoxia group.

### 3.4 Construction of nomogram

**Fig. 5:**
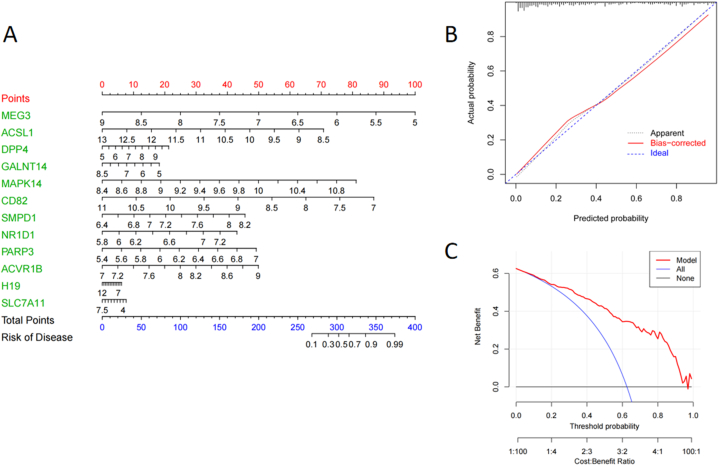
**Establishment of the nomogram** (A) Establishment of a nomogram based on the twelve biomarkers. (B) Calibration curve demonstrating the nomogram's predictive capability. (C) DCA curve of the logistic regression model.

A nomogram predicting the risk of BPD on the basis of the twelve independent marker genes was developed (Fig. 5A). The nomogram calibration curve demonstrated good consistency between prediction and observation groups (Fig. 5B). Based on the DCA curves, the red line was higher compared to the black and gray lines, which implied that BPD patients could profit from the new model if it was utilized for clinical diagnosis (Fig. 5C).

### 3.5 Single-gene GSEA of 12 DE-FRGs

**Fig. 6:**
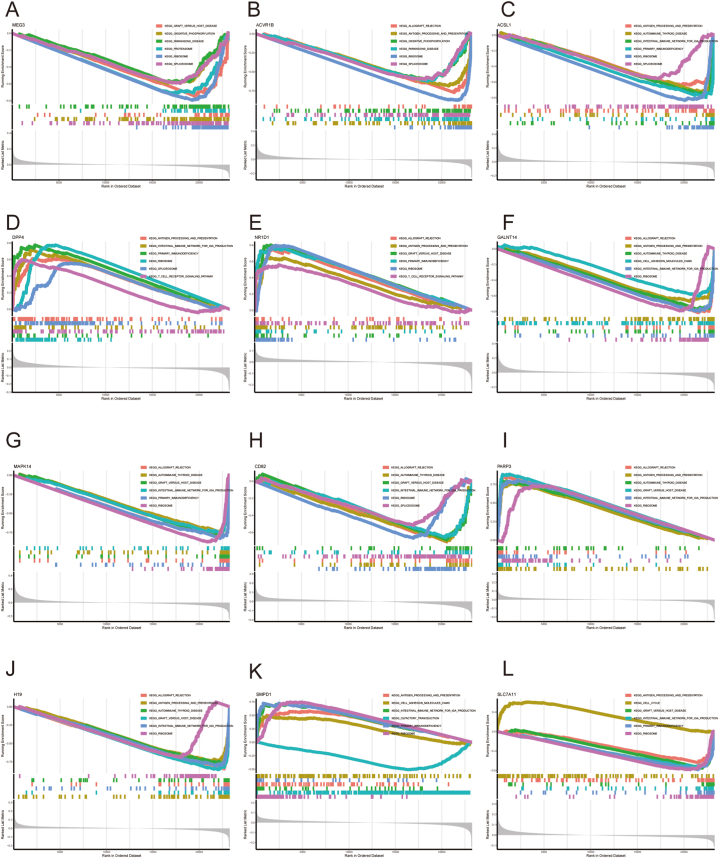
**Single-gene GSEA of 12 DE-FRGs** Single-gene GSEA-KEGG pathway analysis of MEG3 (A), ACVR1B (B), ACSL1 (C), DPP4 (D), NR1D1 (E), GALNT14 (F), MAPK14 (G), CD82 (H), PARP3 (I), H19 (J), SMPD1 (K), and SLC7A11 (L).

Fig. 6A-L presented 6 major enrichment pathways of each marker gene. MEG3 and ACVR1B were closely related to graft versus host disease, oxidative phosphorylation, Parkinson's disease, proteasome, ribosome, and splicesome. ACSL1 was linked to the intestinal immune network for IgA production, immunodeficiency, ribosome, along with splicesome. DPP4 and NR1D1 were associated with primary immunodeficiency, along with the T cell receptor signaling pathway. GALNT14 was related to allograft rejection, cell adhesion molecules cams, and the ribosome. MAPK14 was related to graft versus host disease, and primary immunodeficiency. CD82, PARP3, and H19 were related to allograft rejection, and ribosomes. SMPD1 was related to cell adhesion molecules cams, primary immunodeficiency, etc. SLC7A11 was related to antigen processing and presentation, cell cycle, etc.

### 3.6 Pathways linked to BPD according to marker genes GSVA analysis

**Fig. 7:**
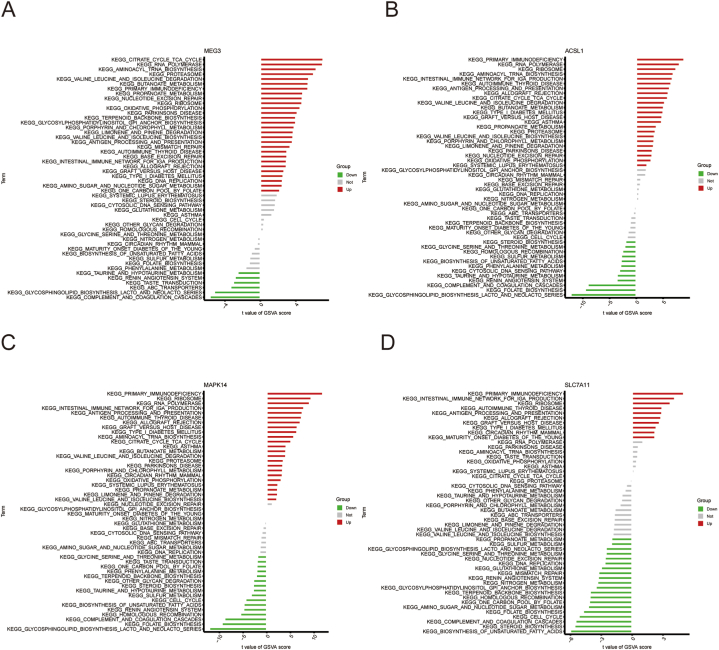
**Pathways according to marker genes GSVA analysis** Low and high expression groups according to the expression levels of each marker gene in combination with GSVA in MEG3 (A), ACSL1 (B), MAPK14 (C), and SLC7A11 (D).

The differential activation pathways in the low and high expression groups were then investigated. The results revealed that highMEG3 expression is associated with citrate cycle TCA cycle, RNA polymerase, aminoacyl tRNA biosynthesis, primary immunodeficiency, as well as oxidative phosphorylation, and low MEG3 expression is associated with ABC transporters, glycosphingolipid biosynthesis of the lacto and neolacto series, etc. High ACSL1 expression is implicated in the activation of primary immunodeficiency, the intestinal immune network for IgA production, etc, whereas the low ACSL1 expression is related with complement and coagulation cascades, etc. Up-regulation of MAPK14 has association with primary immunodeficiency, ribosome, and RNA polymerase, whereas the down-regulation of MAPK14 has relationship with biosynthesis of unsaturated fatty acids, complement and coagulation cascades, folate biosynthesis, etc. A high level of SLC7A11 was associated with primary immunodeficiency, antigen processing, and presentation, allograft rejection, as well as circadian rhythm mammal. Low expression of SLC7A11 is implicated in glutathione metabolism, cell cycle, steroid biosynthesis, and biosynthesis of unsaturated fatty acids, etc. (Fig. 7A–D).

### 3.7 Immune cells infiltration

Recent studies have highlighted a connection between the expression of marker genes and the immune activation response [[30], [31]]. Additionally, accumulating evidence supports the association between BPD and the immune microenvironment [[32], [33]]. To assess the immune cell proportions within the samples, we employed the CIBERSORT algorithm.

**Fig. 8:**
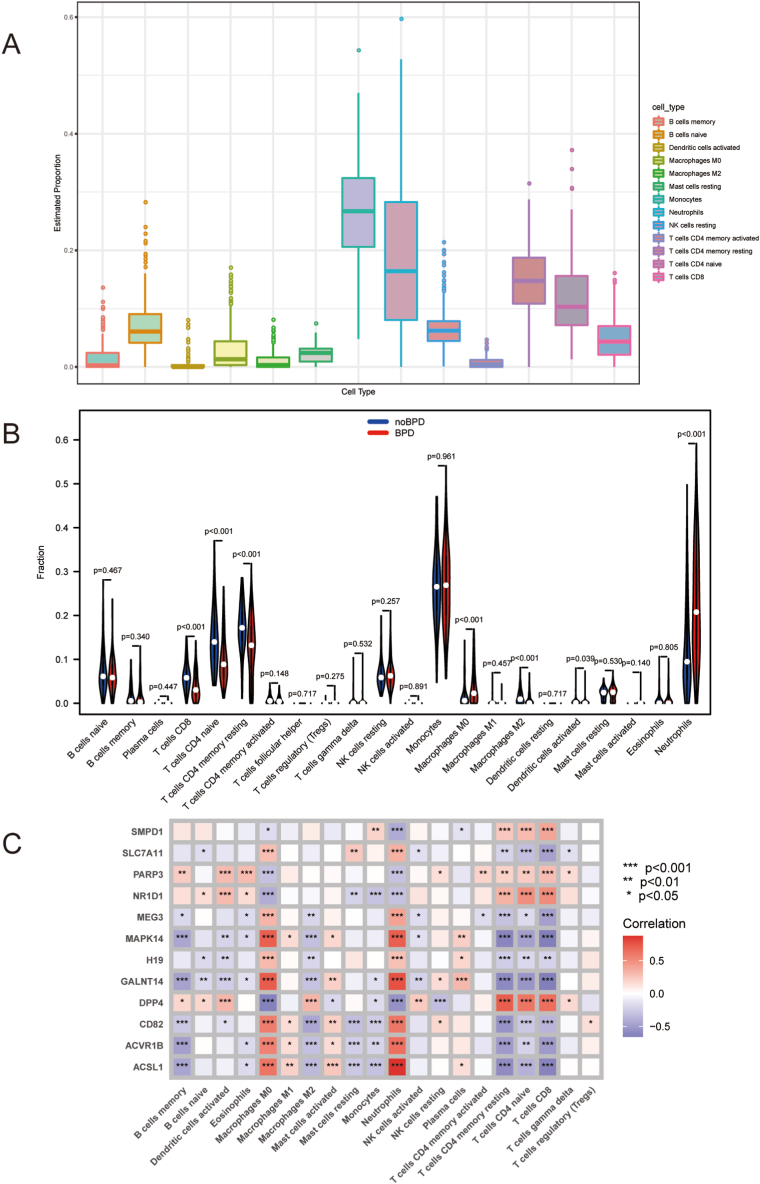
**Analysis of the immune landscape** (A) CIBERSORT analysis was utilized to assess the abundance of immune cell infiltration in BPD. (B) A violin plot displays the differences in immune cell infiltration between BPD and control samples. (C) The correlation plot depicts the relationships among immune cells and the twelve marker genes. Significance levels are indicated as follows: *p < 0.05, **p < 0.01, ***p < 0.001.

In the case of BPD samples, we observed higher proportions of neutrophils, macrophages M0, and monocytes. These differences are depicted in the violin plot, which also showed that BPD samples had a higher fraction of macrophages M0 and neutrophils, along with a lower fraction of T cells CD4 memory resting, T cells CD4 naive, T cells CD8, macrophages M2, and dendritic cells stimulated when compared to control samples (as shown in Fig. 8A and B).

Furthermore, Spearman's correlation analysis revealed significant positive and negative relationships between MAPK14, GALNT14, and various immune cell types, including macrophages M0, neutrophils, T cells CD4 memory resting, T cells CD4 naive, and T cells CD8. Additionally, there were strong positive correlations of CD82, ACVR1B, and ACSL1 with macrophages M0 and neutrophils, while exhibiting strong negative correlations with macrophages M2, mast cells resting, T cells CD4 memory resting, T cells CD4 naive, T cells CD8, and B cells memory.

Moreover, the proportions of T cells CD4 memory resting, T cells CD4 naive, and T cells CD8 displayed positive correlations with the expression of DPP4, while macrophages M0 and neutrophils exhibited negative correlations with DPP4 expression (as illustrated in Fig. 8C). These findings highlight the complex interplay between gene expression, immune cell profiles, and their potential implications in the context of BPD.

### 3.8 Recognition of the possible drugs

**Fig. 9:**
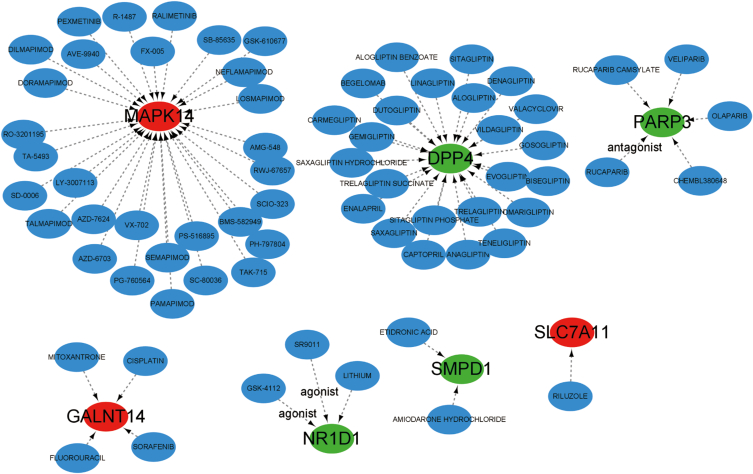
**The drug-gene interaction network** Red ellipses signify the genes that are up-regulated in BPD. Green ellipses represent the genes that are down-regulated in BPD. Blue ellipses denote drugs or compounds. The lines connecting them illustrate drug-gene interactions, indicating the relationships between the drugs and the genes.

DGIdb was used to analyze possible therapeutic agents that could reverse abnormal mean gene expression. As displayed in the drug-gene interaction network (Fig. 9), 30 medicines or molecular compounds, for instance, SEMAPIMOD, DORAMAPIMOD, and TALMAPIMOD were involved in modulating the expression of MAPK14. Moreover, 24 drugs or molecular compounds, consisting of OMARIGLIPTINE, LINAGLIPTIN, and BENMAB, had interactions with DPP4. In addition, there were five drugs or molecular compounds that featured Rucaparib, which acted as a PARP3 antagonist. Furthermore, three drugs or molecular compounds contained both GSK-4112 and SR9011, which worked together to regulate NR1D1.

## 4 Discussion

BPD is a complex disease with an important genetic component. Some researchers claim that various genetic pathways as well as mutations may be related to susceptibility to BPD [34]. Each gene variation that causes dysregulated biological processes in the developing lungs of premature infants may be involved in the development of BPD [35]. Hence a full understanding of BPD pathogenesis will lead to the development of innovative drugs targeting high-risk newborns.

Ferroptosis belongs to an iron-dependent, non-apoptotic type of cell death featured by accumulated lipid reactive oxygen species (ROS) [11]. Ferroptosis is crucial to the biological function process in the following ways: 1) accumulated intracellular iron as well as ROS; 2) stimulated mitogen-activated protein kinase (MAPK) signaling system; 3) decreased cystine (Cys2) uptake and glutathione (GSH) consumption own to inhibition of the Cys2/glutamic acid (Glu) transporter system Xc-transporter on the cell membrane; as well as 4) increased oxidation of nicotinamide adenine dinucleotide phosphate (NADPH) [14,18,36]. Nrf2 effectively inhibits the ferroptosis caused by ischemia/reperfusion and alleviates the acute lung injury (ALI) caused by ischemia/reperfusion. This protective effect is achieved by activating the Nrf2/HIF-1/TF signaling pathway [37]. LPS has the ability to decrease the activity of Beas-2B in the human bronchial epithelial cell line. Fer-1, a ferroptosis inhibitor, has a therapeutic effect on LPS-induced ALI and is capable of reducing pro-inflammatory cytokine levels in bronchoalveolar lavage fluid, thereby suppressing ferroptosis [38]. It has been found that the total GSH is decreased and the oxidized glutathione disulfide (GSSG) is increased in the alveolar epithelium of BPD patients and animal models [39,40]. These findings suggest a potential involvement of iron metabolism and oxidative stress in the development of BPD. It is critical to assess the relationship between ferroptosis and BPD through bioinformatic analysis upon choosing microarray data.

In the current work, we performed an extensive comparison of FRGs expression models in BPD samples with controls. BPD patients were more possible to suffer dysregulated FRGs, underlining the crucial potential of FRGs in BPD. The research recognized twelve different genes related to ferroptosis, containing MEG3, ACSL1, DPP4, GALNT14, MAPK14, CD82, SMPD1, NR1D1, PARP3, ACVR1B, H19, and SLC7A11. The ROC curve showed that the whole twelve genes possessed AUC values > 0.7, which suggested that they were able to differentiate BPD from controls with high precision and specificity. Besides, MEG3, ACSL1 along with DPP4 harbored the top three AUC values. Over activation of MEG3 in rat brain microvascular endothelial cells (RBMVECs) resulted in the suppression of GPX4 expression and ultimately led to ferroptosis triggered by lipid peroxidation [41].

As a part of the long-chain acyl-CoA synthetase family, ACSL1 has been identified to accelerate ferroptosis triggered by α-eleosteric acid [42]. ACSL1 suppression protects cells from MHV-A59 infection with Triacsin C and ferroptosis inhibition [43]. Moreover, it has been reported that DPP4 is up-regulated in the lung parenchyma, epithelial cell surface and vascular endothelium, along with human bronchial fibroblasts [44]. P53 suppresses ferroptosis by directly inhibiting the activity of DPP4 [45]. DPP4 inhibitor Sitagliptin reduces ALI-linked oxidative stress as well as immoderate autophagy via the p62-Keap1-Nrf2 signaling pathway along with Nrf2 nuclear translocation [46].

Additional external verification of the GSE8586 dataset demonstrated that the logistic model based on 10 genes could responsibly predict BPD, broadening the comprehension of BPD diagnosis. Besides, we built a nomogram model for the diagnosis of subtypes of BPD using DE-FRGs. The model demonstrated quite good predictive performance, implying it might be used in clinical contexts.

We then assessed the inter-FRGs relation to clarify the connection between FRGs and BPD. The presence of FRGs interaction in individuals with BPD unveiled that certain FRGs exhibit noteworthy synergistic or antagonistic impacts. New data suggest that immune cells possess an important function in chronic lung disease in premature infants. Based on the CIBERSORT approach, it was observed differences in different immune cells, which contained monocytes, neutrophils, macrophages M0, macrophages M2, T cells CD4 memory resting, T cells CD4 naïve, along with T cells CD8 in BPD and controls. Additionally, MAPK14, GALNT14, CD82, ACVR1B, ACSL1, and DPP4 were linked to multiple immune cells. These results were useful references for further verification. Finally, therapeutic medicines for the marker gene network were examined with the DGIdb database. There was uncertainty about whether our proposed drug was involved, and whether particular routes needed to be further investigated.

It's important to acknowledge some limitations of our study. Firstly, our sample size was relatively small, and as a result, a larger cohort is required to validate and reinforce our findings. Secondly, our study lacked in vivo experiments and had limited in vitro experiments to assess the expression levels of the identified genes. This limitation may have reduced the accuracy and comprehensive understanding of our study. Future research with a larger and more diverse sample size, along with a broader range of experimental approaches, can provide further insights into the role of these genes in BPD. The dataset we analyzed is derived from whole blood, umbilical cords, and mouse whole lung. Validation of these FRGs in human lung tissue is lacked. Additionally, more clinical or experimental assessment is required to validate FRGs expression profiles. The implementation of the predictive model should be certified using a more all-sided set of clinical data. More studies on the potential relationship between FRGs and immunological responses are also essential. Further testing is necessary to build a relationship in the pathology features of BPD.

## 5 Conclusions

The study used bioinformatics methods and databases to build a diagnostic nomogram for BPD using 12 FRGs markers. The study also conducted immune infiltration assays and a drug-gene network. The findings offer novel insights into the molecular mechanisms underlying BPD.

## Data availability statement

All the research data used in this study can be found in GEO. The data could be download at (https://www.ncbi.nlm.nih.gov/geo/; GSE32472, GSE8586, GSE197403, and GSE25286). The original contributions made in this study are incorporated within the article and supplementary materials.

## Author contributions

Zhengyun Hu: Analyzed and interpreted the data.

Chong Liu, Yan Mao: Performed the experiments; Contributed reagents, materials, analysis tools or data; Wrote the paper.

Jianwei Shi, Jinwen Xu, Guoping Zhou, Feng Jiang: Conceived and designed the experiments.

## Funding

The study did not receive any external funding.

## Declaration of competing interest

The authors declare that they have no known competing financial interests or personal relationships that could have appeared to influence the work reported in this paper.

## Figures and Tables

**figs1:**
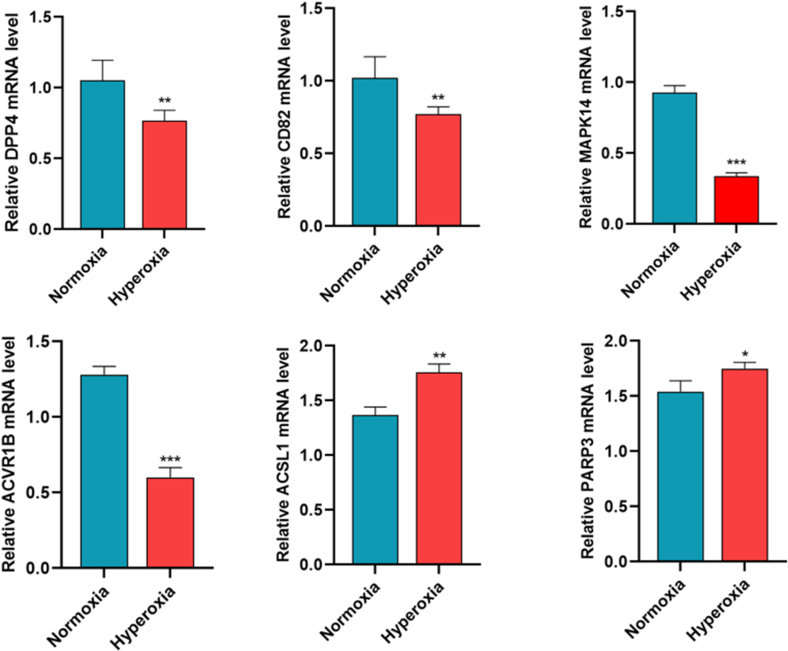


## References

[1] Deng X., Bao Z., Yang X., Mei Y., Zhou Q., Chen A., Yu R., Zhang Y. (2022). Molecular mechanisms of cell death in bronchopulmonary dysplasia. Apoptosis.

[2] Holzfurtner L., Shahzad T., Dong Y., Rekers L., Selting A., Staude B., Lauer T., Schmidt A., Rivetti S., Zimmer K.-P., Behnke J., Bellusci S., Ehrhardt H. (2022). When inflammation meets lung development-an update on the pathogenesis of bronchopulmonary dysplasia. Mol Cell Pediatr.

[3] Wang S.-H., Tsao P.-N. (2020). Phenotypes of bronchopulmonary dysplasia. Int. J. Mol. Sci..

[4] Gilfillan M., Bhandari A., Bhandari V. (2021). Diagnosis and management of bronchopulmonary dysplasia. BMJ.

[5] Principi N., Di Pietro G.M., Esposito S. (2018). Bronchopulmonary dysplasia: clinical aspects and preventive and therapeutic strategies. J. Transl. Med..

[6] Stoll B.J., Hansen N.I., Bell E.F., Walsh M.C., Carlo W.A., Shankaran S., Laptook A.R., Sánchez P.J., Van Meurs K.P., Wyckoff M., Das A., Hale E.C., Ball M.B., Newman N.S., Schibler K., Poindexter B.B., Kennedy K.A., Cotten C.M., Watterberg K.L., D'Angio C.T., DeMauro S.B., Truog W.E., Devaskar U., Higgins R.D. (2015). Eunice kennedy shriver national institute of child health and human development neonatal research network. Trends in Care Practices, Morbidity, and Mortality of Extremely Preterm Neonates, 1993-2012, JAMA.

[7] Truog W.E., Lewis T.R., Bamat N.A. (2020). Pharmacologic management of severe bronchopulmonary dysplasia. NeoReviews.

[8] Roberts K., Stepanovich G., Bhatt-Mehta V., Donn S.M. (2021). New pharmacologic approaches to bronchopulmonary dysplasia. J. Exp. Pharmacol..

[9] Muehlbacher T., Bassler D., Bryant M.B. (2021). Evidence for the management of bronchopulmonary dysplasia in very preterm infants. Children.

[10] Cheng H.-P., Feng D.-D., Yue S.-J., Luo Z.-Q. (2020). [The metabolic networks of ferroptosis and links to lung diseases]. Sheng Li Xue Bao.

[11] Dixon S.J., Lemberg K.M., Lamprecht M.R., Skouta R., Zaitsev E.M., Gleason C.E., Patel D.N., Bauer A.J., Cantley A.M., Yang W.S., Morrison B., Stockwell B.R. (2012). Ferroptosis: an iron-dependent form of nonapoptotic cell death. Cell.

[12] Xu W., Deng H., Hu S., Zhang Y., Zheng L., Liu M., Chen Y., Wei J., Yang H., Lv X. (2021). Role of ferroptosis in lung diseases. J. Inflamm. Res..

[13] Yan H., Zou T., Tuo Q., Xu S., Li H., Belaidi A.A., Lei P. (2021). Ferroptosis: mechanisms and links with diseases. Sig Transduct Target Ther.

[14] Stockwell B.R. (2022). Ferroptosis turns 10: emerging mechanisms, physiological functions, and therapeutic applications. Cell.

[15] Mahoney-Sánchez L., Bouchaoui H., Ayton S., Devos D., Duce J.A., Devedjian J.-C. (2021). Ferroptosis and its potential role in the physiopathology of Parkinson's Disease. Progress in Neurobiology.

[16] Li H., Lin Y., Zhang L., Zhao J., Li P. (2022). Ferroptosis and its emerging roles in acute pancreatitis. Chin Med J (Engl)..

[17] Yin X., Zhu G., Wang Q., Fu Y.D., Wang J., Xu B. (2021). Ferroptosis, a new insight into acute lung injury. Front. Pharmacol..

[18] Li Y., Yang Y., Yang Y. (2022). Multifaceted roles of ferroptosis in lung diseases. Front. Mol. Biosci..

[19] Qu M., Zhang H., Chen Z., Sun X., Zhu S., Nan K., Chen W., Miao C. (2021). The role of ferroptosis in acute respiratory distress syndrome. Front. Med..

[20] Qiang Z., Dong H., Xia Y., Chai D., Hu R., Jiang H. (2020). Nrf2 and STAT3 alleviates ferroptosis-mediated IIR-ALI by regulating SLC7A11. Oxid. Med. Cell. Longev..

[21] Lingappan K., Srinivasan C., Jiang W., Wang L., Couroucli X.I., Moorthy B. (2014). Analysis of the transcriptome in hyperoxic lung injury and sex-specific alterations in gene expression. PLoS One.

[22] Yan W., Jiang M., Zheng J. (2020). Identification of key pathways and differentially expressed genes in bronchopulmonary dysplasia using bioinformatics analysis. Biotechnol. Lett..

[23] Zhang L., Wang P., Shen Y., Huang T., Hu X., Yu W. (2022). Mechanism of lncRNA H19 in regulating pulmonary injury in hyperoxia-induced bronchopulmonary dysplasia newborn mice. Am. J. Perinatol..

[24] Li J., Lu K., Sun F., Tan S., Zhang X., Sheng W., Hao W., Liu M., Lv W., Han W. (2021). Panaxydol attenuates ferroptosis against LPS-induced acute lung injury in mice by Keap1-Nrf2/HO-1 pathway. J. Transl. Med..

[25] Faul F., Erdfelder E., Buchner A., Lang A.-G. (2009). Statistical power analyses using G*Power 3.1: tests for correlation and regression analyses. Behav. Res. Methods.

[26] Zhou N., Yuan X., Du Q., Zhang Z., Shi X., Bao J., Ning Y., Peng L. (2023). FerrDb V2: update of the manually curated database of ferroptosis regulators and ferroptosis-disease associations. Nucleic Acids Res..

[27] Zhou Y., Zhou B., Pache L., Chang M., Khodabakhshi A.H., Tanaseichuk O., Benner C., Chanda S.K. (2019). Metascape provides a biologist-oriented resource for the analysis of systems-level datasets. Nat. Commun..

[28] Le T., Aronow R.A., Kirshtein A., Shahriyari L. (2021). A review of digital cytometry methods: estimating the relative abundance of cell types in a bulk of cells. Brief Bioinform.

[29] Cotto K.C., Wagner A.H., Feng Y.-Y., Kiwala S., Coffman A.C., Spies G., Wollam A., Spies N.C., Griffith O.L., Griffith M. (2018). DGIdb 3.0: a redesign and expansion of the drug-gene interaction database. Nucleic Acids Res..

[30] Y. Mao, J. Xu, X. Xu, J. Qiu, Z. Hu, F. Jiang, G. Zhou, Comprehensive analysis for cellular senescence-related immunogenic characteristics and immunotherapy prediction of acute myeloid leukemia, Front. Pharmacol.. (n.d.) 15. 10.3389/fphar.2022.987398 PMC9548549 36225590

[31] Y. Mao, Z. Hu, X. Xu, J. Xu, C. Wu, F. Jiang, G. Zhou, Identiﬁcation of a prognostic model based on costimulatory molecule-related subtypes and characterization of tumor microenvironment inﬁltration in acute myeloid leukemia, Front. Genet.. (n.d.) 14. 10.3389/fgene.2022.973319 PMC9437340 36061194

[32] Chen L., Shi C., Zhou G., Yang X., Xiong Z., Ma X., Zhu L., Ma X., Mao Y., Hu Y., Wang J., Tang X., Bao Y., Ma Y., Luo F., Wu C., Jiang F. (2023). Genome-wide exploration of a pyroptosis-related gene module along with immune cell infiltration patterns in bronchopulmonary dysplasia. Front. Genet..

[33] Tao Z., Mao Y., Hu Y., Tang X., Wang J., Zeng N., Bao Y., Luo F., Wu C., Jiang F. (2023). Identification and immunological characterization of endoplasmic reticulum stress-related molecular subtypes in bronchopulmonary dysplasia based on machine learning. Front. Physiol..

[34] Higano N.S., Ruoss J.L., Woods J.C. (2021). Modern pulmonary imaging of bronchopulmonary dysplasia. J. Perinatol..

[35] Gilfillan M., Bhandari V. (2022). Moving bronchopulmonary dysplasia research from the bedside to the bench. Am. J. Physiol. Lung Cell Mol. Physiol..

[36] Zheng D., Liu J., Piao H., Zhu Z., Wei R., Liu K. (2022). ROS-triggered endothelial cell death mechanisms: focus on pyroptosis, parthanatos, and ferroptosis. Front. Immunol..

[37] Dong H., Qiang Z., Chai D., Peng J., Xia Y., Hu R., Jiang H. (2020). Nrf2 inhibits ferroptosis and protects against acute lung injury due to intestinal ischemia reperfusion via regulating SLC7A11 and HO-1. Aging (Albany NY).

[38] Liu P., Feng Y., Li H., Chen X., Wang G., Xu S., Li Y., Zhao L. (2020). Ferrostatin-1 alleviates lipopolysaccharide-induced acute lung injury via inhibiting ferroptosis. Cell. Mol. Biol. Lett..

[39] Jia D., Zheng J., Zhou Y., Jia J., Ye X., Zhou B., Chen X., Mo Y., Wang J. (2021). Ferroptosis is involved in hyperoxic lung injury in neonatal rats. J. Inflamm. Res..

[40] Fan R., Sui J., Dong X., Jing B., Gao Z. (2021). Wedelolactone alleviates acute pancreatitis and associated lung injury via GPX4 mediated suppression of pyroptosis and ferroptosis. Free Radic. Biol. Med..

[41] Chen C., Huang Y., Xia P., Zhang F., Li L., Wang E., Guo Q., Ye Z. (2021). Long noncoding RNA Meg3 mediates ferroptosis induced by oxygen and glucose deprivation combined with hyperglycemia in rat brain microvascular endothelial cells, through modulating the p53/GPX4 axis. Eur. J. Histochem..

[42] Beatty A., Singh T., Tyurina Y.Y., Tyurin V.A., Samovich S., Nicolas E., Maslar K., Zhou Y., Cai K.Q., Tan Y., Doll S., Conrad M., Subramanian A., Bayır H., Kagan V.E., Rennefahrt U., Peterson J.R. (2021). Ferroptotic cell death triggered by conjugated linolenic acids is mediated by ACSL1. Nat. Commun..

[43] Xia H., Zhang Z., You F. (2021). Inhibiting ACSL1-related ferroptosis restrains murine coronavirus infection. Viruses.

[44] Zhang T., Tong X., Zhang S., Wang D., Wang L., Wang Q., Fan H. (2021). The roles of dipeptidyl peptidase 4 (DPP4) and DPP4 inhibitors in different lung diseases: new evidence. Front. Pharmacol..

[45] Kang R., Kroemer G., Tang D. (2019). The tumor suppressor protein p53 and the ferroptosis network. Free Radic. Biol. Med..

[46] Kong L., Deng J., Zhou X., Cai B., Zhang B., Chen X., Chen Z., Wang W. (2021). Sitagliptin activates the p62-Keap1-Nrf2 signalling pathway to alleviate oxidative stress and excessive autophagy in severe acute pancreatitis-related acute lung injury. Cell Death Dis..

